# Product Design by Additive Manufacturing for Water Environments: Study of Degradation and Absorption Behavior of PLA and PETG

**DOI:** 10.3390/polym13071036

**Published:** 2021-03-26

**Authors:** Daniel Moreno Nieto, María Alonso-García, Miguel-Angel Pardo-Vicente, Lucía Rodríguez-Parada

**Affiliations:** Mechanical Engineering and Industrial Design Department, Engineering Faculty, University of Cádiz, Puerto Real, 11510 Cádiz, Spain; daniel.moreno@uca.es (D.M.N.); maria.alonso@uca.es (M.A.-G.); miguelangel.pardo@uca.es (M.-A.P.-V.)

**Keywords:** additive manufacturing, degradation, PLA, PETG, absorption, product design

## Abstract

Additive manufacturing technologies are shifting from rapid prototyping technologies to end use or final parts production. Polymeric material extrusion processes have been broadly addressed with a specific definition of all parameters and variables for all different of technologies approaches and materials. Recycled polymeric materials have been studied due to the growing importance of the environmental awareness of the contemporary society. Beside this, little specific research has been found in product development applications for AM where the printed parts are in highly moisture environments or surrounded by water, but polymers have been for long used in such industries with conventional manufacturing approaches. This work focuses on the analysis and comparison of two different additively manufactured polymers printed by fused filament fabrication (FFF) processes using desktop-size printers to be applied for product design. The polymers used have been a recycled material: polyethylene terephthalate glycol (PETG) and polylactic acid (PLA). Degradation and water absorption behaviors of both materials are presented, analyzed and discussed in this paper, where different samples have been immersed in saturated solutions of water with maritime salt and sugar together with a control sample immersed in distilled water. The samples have been dimensionally and weight-controlled weekly as well as microscopically analyzed to understand degradation and absorption processes that appear in the fully saturated solutions. The results revealed how the absorption process is stabilized after a reduced number of weeks for both materials and how the degradation process is more remarked in the PLA material due to its organic nature.

## 1. Introduction

Additive manufacturing (AM) processes are increasing their visibility in the industrial scene, shifting from prototyping tools to the production of final parts or tools. This is due to the improved reliability of the processes as well as improvements and development in the materials field. 

The American Society for Testing and Materials (ASTM) formed the ASTM Committee F42 in 2009. This committee presents a classification of seven different technologies using different energy sources or material feedstock, among them material extrusion (ME) processes [1]. AM field is experiencing an exponential growth that implies the development of new technologies together with new successful cases and applications. These technologies are based in the construction of almost any kind of geometrical shapes with a layer-by-layer approach that permits the construction of specific parts or custom-made tools with great reductions of times and cost-savings [2]. It can be understood that its use is recommendable for certain parts or products with specific needs, referring to complex shapes and custom demands cohabitating AM technologies with other existing subtractive technologies.

This work focusses on fused filament fabrication (FFF) technologies, which is the most widespread technology classified in ME processes according to the ASTM. Scott Trump first developed the original Fused Deposition Modelling™ (FDM™) technology in 1988, and its patent expired in the year 2009. This has permitted the growth of different projects and brands that nowadays commercialize this equipment in a wide array of specifications and components going from domestic use to industrial applications.

The AM research field has exponentially grown in the last few years with a strong diversification of research topics. Different publications can be found related to the anisotropic mechanical properties of different three-dimensional (3D) printed materials [3,4], together with rheological and thermal characterization [5], giving great insights into the products and parts performance whilst printing and after print. This helps to prevent thermomechanical distortions and, consequently, the decrease in physical properties during the lifetime of printed parts [6]. Material development plays an important role when trying to achieve the optimum properties for the processing and in-service behavior of printed products. For this purpose, material developers are researching the application of different types of additives of micro- and nanonature [7] that aim to improve the process and material performance, such as microfibers, microcrystal spheres, or continuous fibers [8]. Design for additive manufacturing (DfAM) is also a common field of research where different approaches are generally addressed, from design guides and recommendations to specific study cases with specific tools especially developed for AM process, such as topology optimization (TO) or lattice design that can enhance the benefits of the freeform design capabilities of these technologies [9]. Numerical finite element simulation of FFF specimens is also a current challenge, which aims to create standardized test methods for specific products, materials, and part behavior prediction virtually [10,11]. Mechanical characterization with physical testing is also an important topic in the field of AM with different methods already considered as standards, to highlight García-Domínguez et al. work where a full description and a comparative analysis of the existing standards are presented [12]. Another field of study is postprocessing, physical or chemical approaches which have been reported from sanding or milling [13] to different chemical baths [14].

Few references regarding the analysis of 3D printed parts or specimens in high-moisture environments have been found in the literature. To highlight the research of Algarni [15], which presents a study describing the effect of moisture and raster orientation in the mechanical properties of polylactic acid (PLA) specimens. Results show 10% of moisture in specific raster orientations improves mechanical and strength properties. Ayrilmis et al. [16] presented the effect of layer thickness on the water absorption rates of PLA with wood flour materials together with the mechanical properties, pointing out how the water absorption increases with the layer height. Vicente et al. [17] studied the effect of different surface coatings in the water absorption and mechanical properties, concluding that different polyurethane coatings reduce water absorption rates and increase the mechanical properties of the materials. In addition, Kakanaru et al. [18] studied the water absorption rates and degradation times of PLA, ABS (acrylonitrile butadiene styrene), and PLA with silicon carbide, revealing that the addition of 20% of Si–C particles increases the degradation times as well as improving the mechanical properties of the materials. Kariz et al. [19] presented the study of different wood powder mixtures in a PLA matrix, defending that the higher wood content increases the water absorption rate and decreases the mechanical properties of the materials. Kwon et al. [20] studied the changes on the dimensional stability of parts printed with the selective laser sintering (SLS) technology of polyamide 12 when exposed to humidity cycles that affects dimensional stability in low percentages. Regarding the hydrolytic degradation of biopolymers like PLA, it has been presented by the fact that the polymeric chains rearrangement that happens in the printing process increases its susceptibility to water [21] and accelerate its decomposition over time [22,23].

Sustainability is also an important trend in the field of material selection or development for ME processes, as is the case with FFF technology, especially in view of global environmental problems and the fact that the global production of plastics will achieve 3.4 billion tons in 2050 according to the World Bank statistics [24]. As this technology is growing in demand, different solutions are taken into consideration for its further application in FFF technologies. Biopolymers like PLA are one of the most used in the domestic scene for 3D printing but also recycling and reuse of already printed materials is everyday more present, and some commercial brands have already dispensed materials that haven been recycled from previous printed parts or come from other industrial applications. This is the case of polyethylene terephthalate glycol (PETG), a mainstream recycled material used in the packaging industry and also used in the AM industry due to its processability together with its good behavior against corrosion and chemical and physical resistance [4]. Other industries using PLA and PETG are as following: medical due to the biocompatible characteristics of these materials [25], packaging [26,27] and maritime fields [28,29]. This research can be applied to the maritime and packaging industries due to the characteristics of product types and part requirements in these environments.

Amongst the new technologies, AM pays special attention to product design in the industry throughout the entire product life cycle. For instance, packaging products can demand the application of waterproof 3D printed parts for high-moisture environments, giving protection during transport, generating fast supply spare parts and offering new features or qualities [30] in highly-moisture environments that frequently contain sugar.

Another remarkable field with high-moisture environments is, as mentioned above, the naval and maritime industry. This industry also is focusing on understanding the change of properties of materials exposed to salty water in seas and oceans and regular water in lakes. For this reason, it will provide critical information for successful parts developments that guarantee the normal behavior in the aggressive environment. Together with the need of watertight products for specific applications. Some products that already have been printed include a developed toilet cabin from the Spanish national Shipyard [31], a submarine prototype by the US army [32] and a recently developed racing boat by an Italian company that is planning to cross the ocean this year which is part of the so-called Livrea project [33]. 

In this sense, the use of AM for products in water environments is an almost unexplored area that can offer customized designs as well as testing short series without the need of tooling and with energy-savings from the product’s life cycle. This technology can be beneficial for the generation of new product typologies that allow contact with liquids. It should be added that, just as 3D printing is booming, the creation of customized and parameterized products in water environments is a field that has yet to be explored and can offer great advantages.

In this context, understanding PLA and PETG behavior and analyzing their water absorption and degradation properties for future industrial applications have motivated the present research, which will lead the authors to develop functional prototypes or specific 3D printed components or products for specific needs and contexts that may provide useful solutions for their use when required. Of the two industries analyzed, a case study of product design for marine environments, specifically a buoy, has been developed in this work.

## 2. Materials and Methods

The selected materials for this study were as follows: a PETG and a sugarcane-based PLA from the company Smartmaterials™ (Jaén, Spain). Both materials were filaments with 1.75 mm in diameter and in natural color. First, PETG is a thermoplastic polyester that does not crystallize when it is heated up. PETG is also compatible with the human body having a good chemical resistance and transparency. The material with a density of 1.27 g/cm^3^ presents a low water absorption index of 0.16% together with good mechanical properties [34]. Second, PLA is a thermoplastic biopolymer with a natural origin and a density of 1.24 g/cm^3^ and a high water absorption rate around 1% with excellent mechanical properties [35]. The cost of this materials is around €15/kg for PLA and €20/kg for PETG.

All the specimens of this research were printed using a Witbox One from Bq™ (Madrid, Spain) and a Zortrax™ (Olsztyn, Poland) desktop printer. Two different experiments were developed, based on which the research was structured: the degradation and water absorption. These experiments were carried out with square-shaped specimens with dimensions of 30 mm × 30 mm × 3 mm. A total of 45 specimens were printed on each of the materials. Fifteen samples of each material were immersed in three different solutions, distilled water and two fully saturated solutions of distilled water, maritime salt, and white sugar, According to [36]. The selection of these two solutes was based on their natural existence in some of the most remarkable application fields such as the maritime and food packaging industries.

**Figure 1:**
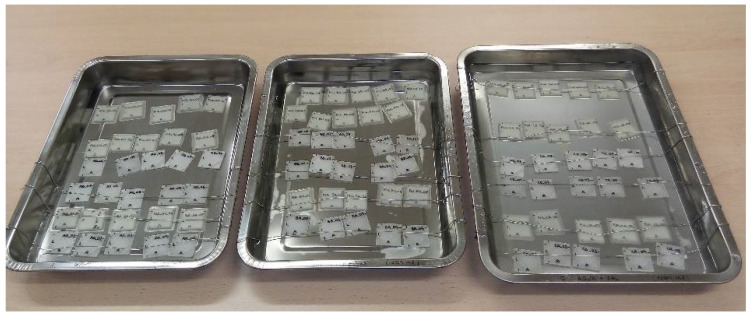
Experiment setup.

The calculations were based on the fact that a fully saturated water salt solution needed 39 g of salt for every 100 mL at 20 °C (28.05% on weight). The sugar fully saturated dissolution needed 133 g for every 100 mL of water at 20 °C (57.08% on weight). The sugar solution needed 2.592 L of distilled water and 3.447 kg of sugar, and the salt solution needed 2.595 L of distilled water and 1.082 kg of salt. Figure 1 shows the experimental setup where a tin wire was used to prevent the specimens’ flotation. The printing parameters selected to print the aforementioned specimens are reflected in Table 1, following the material supplier indications. Thermogravimetric analysis (TGA) determined the degradation temperature of the samples, as the degradation temperature range of PETG is over 350 °C [37].

The microscopic analysis was performed using an optical microscope with a 500 nm resolution (Rohs™), connected to a computer that enabled the researchers to record the images used for the analysis. The weight measurements were accomplished with a Pioneer Ohaus analytical balance with a precision of ±0.001 g. The dimensional measurements were done with a caliper with a precision of ±0.01 mm.

The experimental procedure was defined according to the ISO standards [36,38,39]. The experiments lasted 10 weeks for degradation tests, but for the absorption test, it was only concluded once the weight of the specimens got stable. The measurements were done weekly at the same time everyday for the degradation test. The measurements were conducted daily during the first week of the absorption test and later once a week. The environmental conditions were kept constant in a controlled environment of 20 °C and 50% of humidity for all samples, and the solution were agitated once a day by changing the solution weekly.

For the absorption test, each week five samples were extracted from the 15 samples immersed at the beginning. This samples were cleaned to remove any possible solutes attached to the surfaces of each sample (sugar or salt). The samples were dried with blotting paper. The samples were weighed and measured, after which they were placed in the solution to continue the process. 

**Figure 2:**
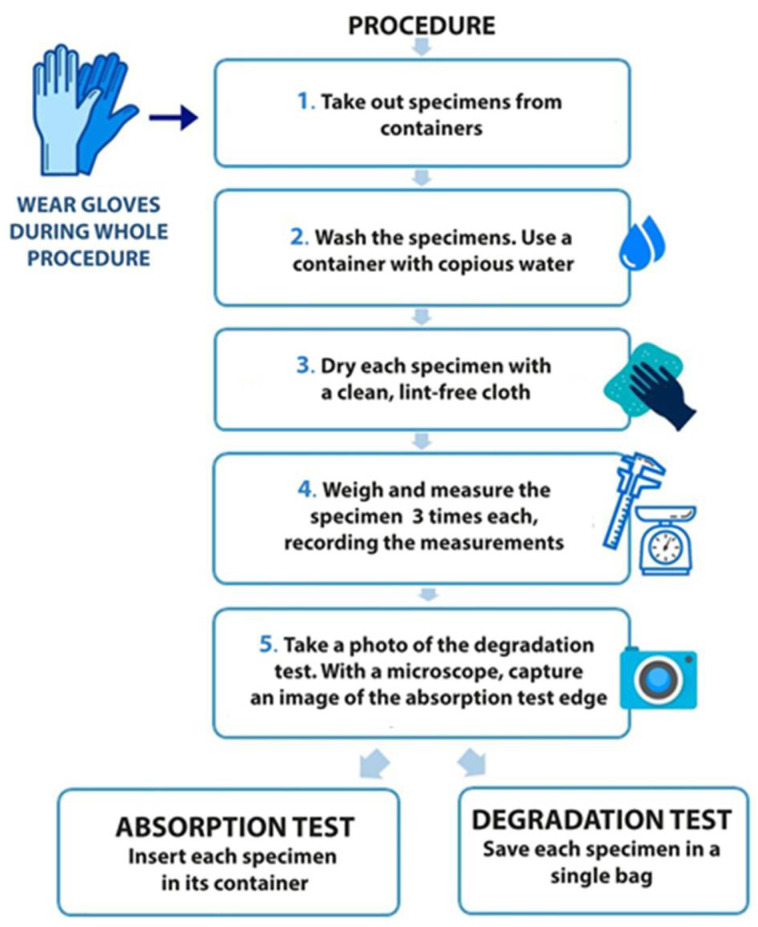
Experimental procedure for measurements.

Ten of the immersed samples were used for the degradation test. Each week, one of the samples was removed, and the same drying procedure was followed as for the absorption samples. In this case, as mentioned above, only one specimen was visually analyzed. A photograph was also taken in accordance with the standard ISO 175:2001 [39]. After visual inspection, the sample was observed under an optical microscope and kept in a plastic bag out of the solution. This procedure is depicted in Figure 2. The specimens were always handled with stainless-steel forceps.

## 3. Results and Discussion

The procedure to obtain the results consisted of two experimental blocks that helped to understand the behavior of the 3D printed parts. Firstly, the water absorptions of specimens in all different solutions have been quantified. Secondly, the degradation of the samples when immersed in fully saturated solutions of maritime salt and white sugar was analyzed. The results have been compared with those of the samples immersed in distilled water with no solute for the degradation and absorption specimens. This data provided useful insights applied in product development cases, presented as a conclusion of this work.

### 3.1. Water Absorption Test

The water absorption test was specifically designed to observe the continued evolution in size and weight, according to the standard ISO 62:2008 [36]. Five different samples of PETG and five of PLA were printed and immersed in three different solutions of distilled water (one of distilled water alone and the two others with fully saturated solutions of maritime salt and white sugar). All the specimens were measured and weighted weekly by removing them from the buckets according to the previously explained procedure and registering their specific dimensions and weights. 

**Figure 3:**
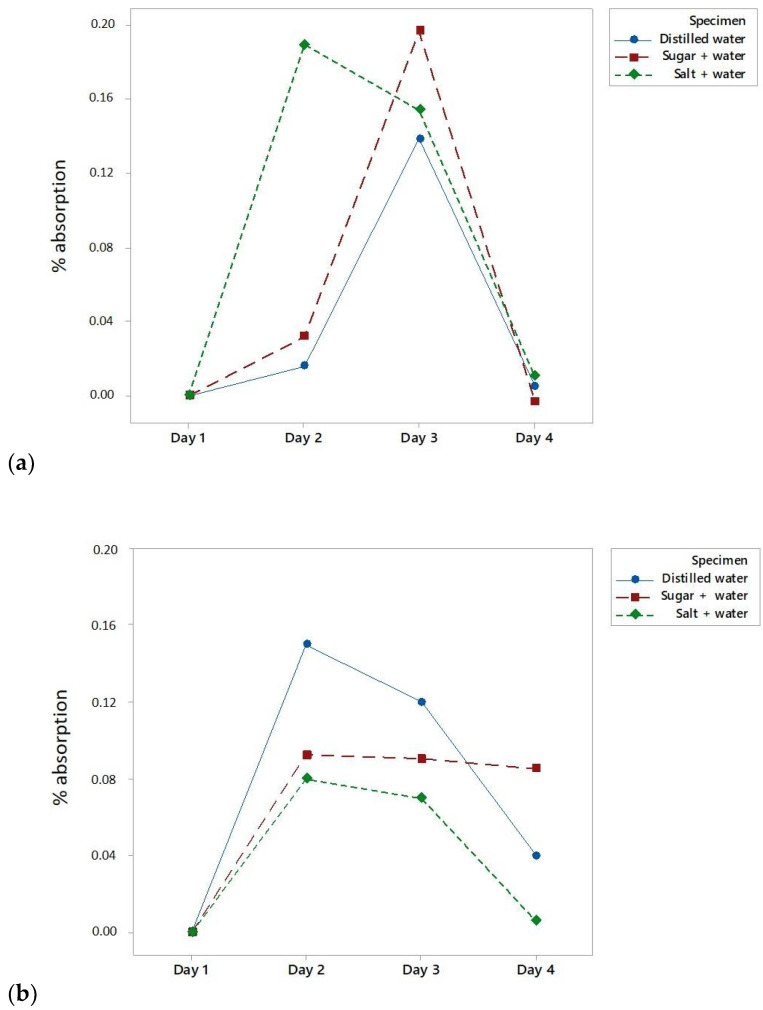
Water absorption for the immersed specimens: (**a**) PLA values; (**b**) PETG values.

It has been observed that for both materials the main absorption occurred in the first two or three days of the experiment. The graphs (Figure 3) reflect the percentage of weight variation from the previous day. The PETG and PLA samples had the highest amount of weight increase in day 2 and day 3, respectively.

It was deduced from previous graphs that the highest amount of liquid for all solutions happened in the very first days, probably because the liquid rapidly flowed inside the specimens through all the defects, micro holes, and the bonding defects of the external layers. In addition, it is interesting to remark that for PLA the highest absorbed solution was the fully saturated solution of sugar and that probably together with the water absorption the organic nature of PLA and sugar facilitates an adhesion of sugar particles to the printed specimen. For PETG, the control sample of pure distilled water had the highest absorption rate. This revealed the capacity of this material to repel any kind of substances aligned with its use in the packing industry [40,41,42,43]. 

**Figure 4:**
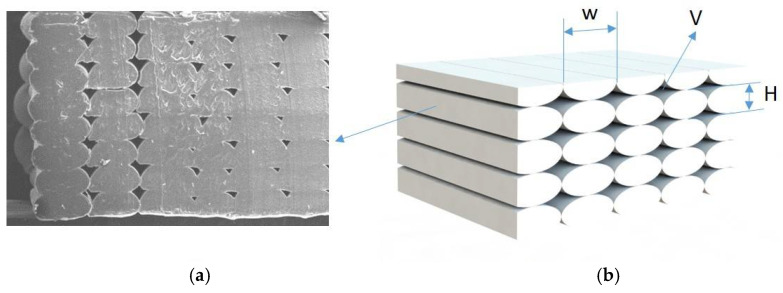
(**a**) Example of three-dimensional (3D) printed part microestructure by SEM. (**b**) Schematic of a 3D printed microestructure observed by SEM where H is the layer hight, W is the road width, and V is the void between roads.

**Figure 5:**
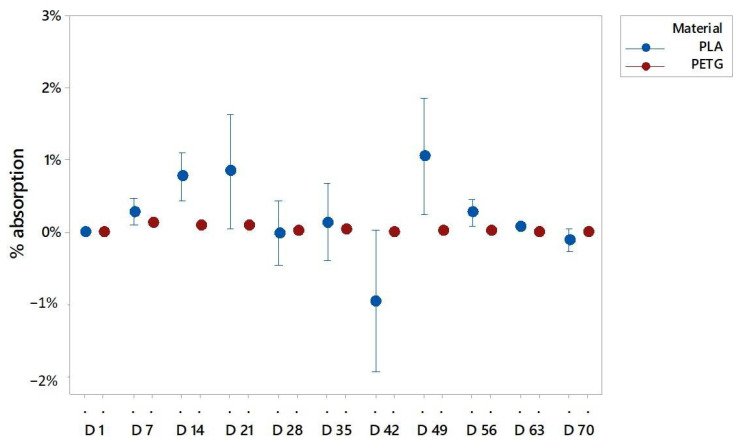
Water absorptions of PLA and PETG specimens in distilled water, where *D* is the day of the evaluation of absorption.

**Figure 6:**
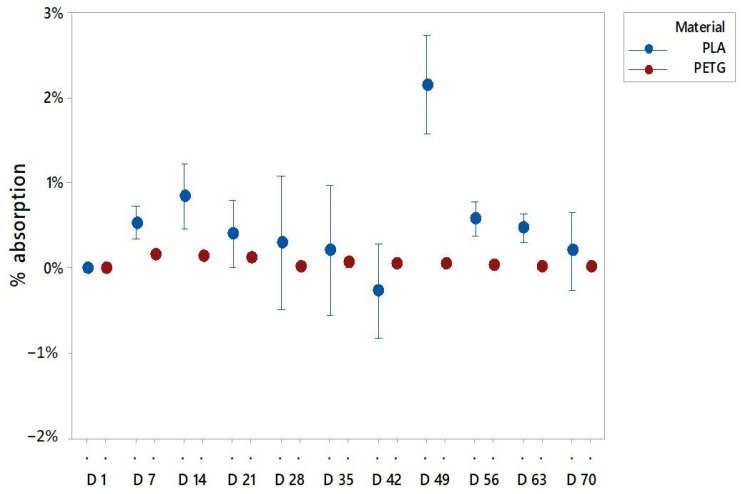
Water absorptions of PLA and PETG specimens in a fully saturated solution with distilled water and sugar, where *D* is the day of the evaluation of absorption.

The most relevant value to explain the water absorption is the weight. Figure 4, Figure 5 and Figure 6 present the mean of the percentual weight increase. The key factor in understanding water absorption is the weight stabilization according to the standards.

All PLA solutions presented weight reductions due to its organic nature and degradation procceses like hydrolisis, as described later on. Disolving the external layers permitted the fluid to flow in and increased the specimen weight, as percieved in the graphs with an increase of weight untill it stabilized.

**Figure 7:**
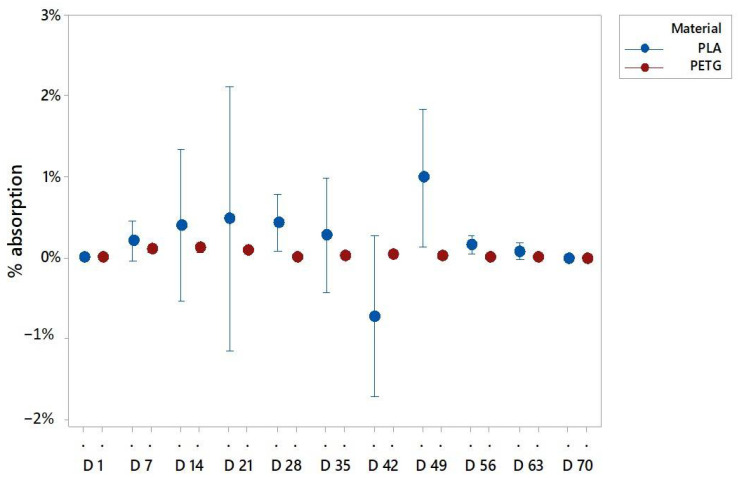
Water absorptions of PLA and PETG specimens in a fully saturated solution with distilled water and salt, where d is the day of the evaluation of absorption.

In particular, the PLA samples immersed in pure distilled water stabilized in weight before week 8 (Figure 5). The PLA in sugar solutions had a longer period to be stabilized, probably due to the easiness of the sugar to adhere to the specimen surface that can block the water integration on the matrix. Then, the sugar was also removed from the surface while being dried to allow the abortion until week 9 (Figure 6). For PLA in salt solutions the weights stabilized at week 3, but after 5 weeks some weight variations were recorded (Figure 7). This is due to the organic nature of the material that may experience in water environments some degradation through hydrolysis which may dissolve the external layers of the specimens. This finding revealed some gaps or cavities inherent to AM processes (Figure 4), according to [44,45], where suddenly the water can flow and increase the specimen weight. On day 49, a remarkable change in the percentual weight variation regarding the PLA samples was presented. This is probably due to the degradation of an internal extrusion liberating internal voids, and this may lead to an increase of the water absorption percentual rate.

In the case of PETG, different results of stabilization were obtained, depending on the solution. It was observed that weights tended to stabilize after week 8 for the specimens immersed in pure distilled water (Figure 5). For the specimens immersed in the sugar solution, it is in week 9 when the weights got stabilized (Figure 6), and for the PETG specimens immersed in the salt solution the weights got stable at week 7 (Figure 7). It should be highlighted that the sugar solution presented a longer period to stabilize its weight. This may be due to the adhesion of sugar molecules to the surface that can block the cavities through which the solution must flow inside the specimens. In addition, the variations in the weights collected may also be due to the drying process, because the weight value may be affected by the removal of water and sugar particles adhering to the surface. In all cases, PETG results remained stable from the first few weeks due to its chemical stability, making it suitable for its application in product and part design. 

As it can be observed in the graphs, both materials behaved similar, presenting slightly higher values of absorption in sugar-saturated solutions and a less stable behavior in salt-saturated solutions. 

According to the literature [46], PETG specimens absorption rates are around 0.15%, and the tested specimens have presented a weight variation rate of 0.3%, compared PLA specimens which had a bigger increase in mean weight of 2.5%, while absorption rates in the literature are around under the 1%. These variations can be explained, because the layered structure of the printed specimens facilitated the absorption due to printed defects and the discontinuity of the external surfaces, together with the adhesion of the salt and sugar molecules from the saturated solutions. Comparing the behaviors of both materials it is highlighted that beside the fact that the stabilization of water absorption times is similar in both materials, PETG specimens behaved more stable due to its high degree of crystallinity. 

It should be also remarked that the 3D printed parts did not behave as the original source material and no 3D printed parts obtained with FFF were homogenious with isotropic properties. Once printed, their behaviour should be understood like microstructures with internal voids and anisotropic properties; therefore, water absorption rates will change compared to the original material data sheet values. In this case, the absorption rates not only from the material but also from the fluid inmersion in the microstructure were considered, as shown in Figure 4.

### 3.2. Degradation Test

The degradation test was performed for 10 weeks, where a single specimen was measured, weighted and observed weekly with optical microscopy. This experiment was executed according to the standard ISO 9227 [38]. All the specimens were identified and immersed in the three different solutions.

**Figure A1:**
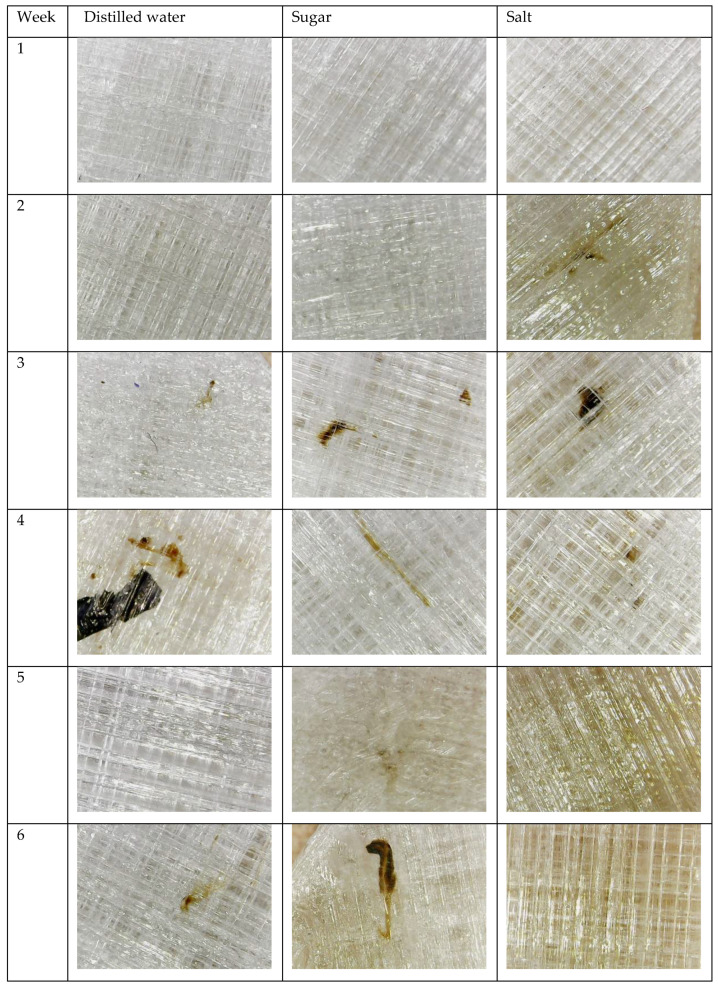

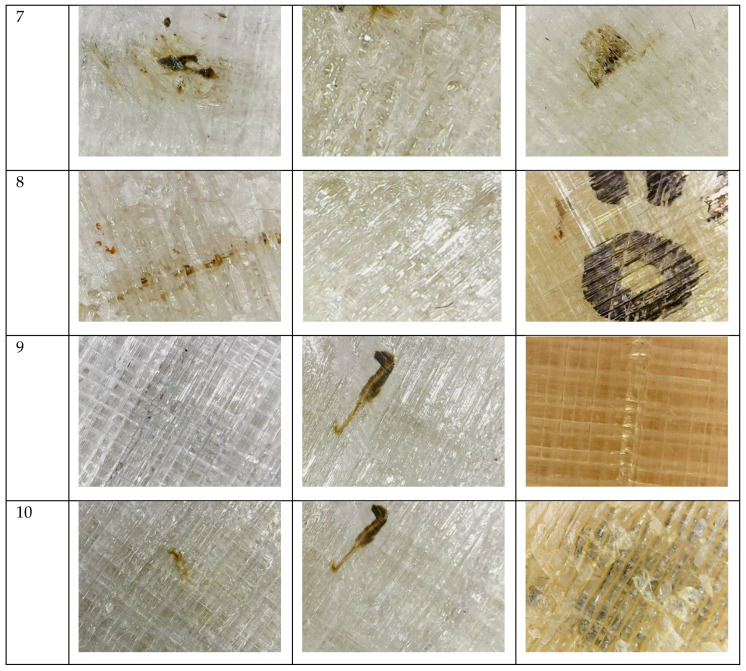
Degradation of PETG.

For PETG specimens, no significant change was observed until week 4 of the experiment (Figure A1 in Appendix A). The distilled water solution reveals small brownish dots appeared in week 4. In week 5, the dots expanded to the surface and the interior of the specimen. In the following week’s specimens, borders started to deteriorate. The solution fully saturated with sugar on week 4 also presented small stains and appeared in the specimens. In week 5, this stain turned to a subtle beige tone without increasing in size. In the following weeks, the growth of this stain was slower than in the previous specimens. The PETG specimens immersed in the salty solution presented changes after two weeks, turning slightly to a yellowish tone. In addition, small brownish dots appeared. After week 3, the material started to turn transparent. This behavior remained in the following weeks, and the solution became more transparent every week. The entire specimen became more transparent; this may be due to a change in the crystallinity of the polymer. 

**Figure A2:**
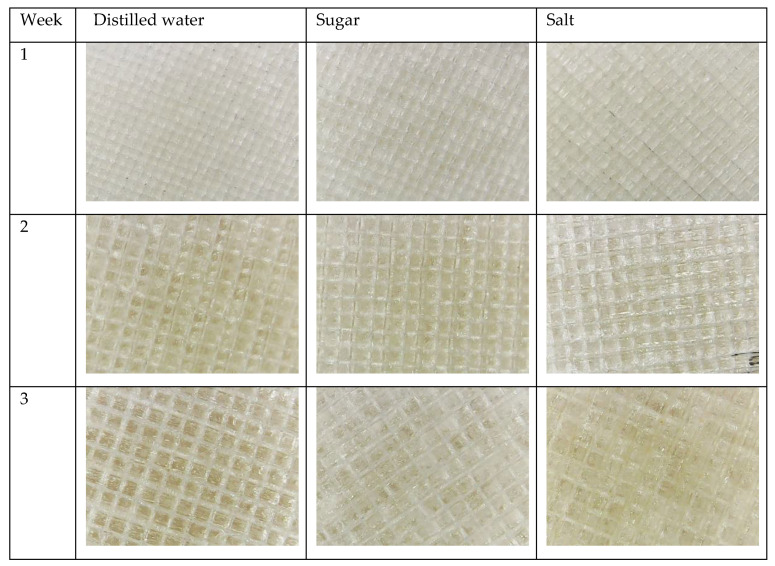

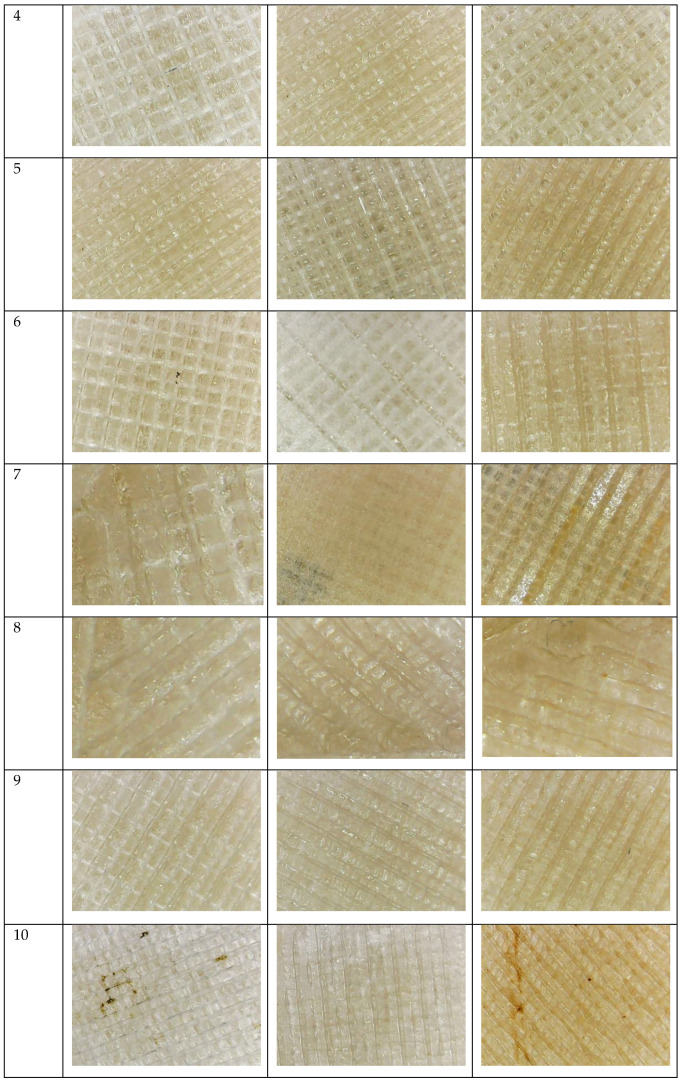
Degradation of PLA.

For the PLA specimens immersed in salt and sugar, after week 2, first degradation signals were observed, due to its lack of resistance to the molecules in the solutions and organic nature. All samples turned slightly yellow colored compared to the reference sample. In week 4, the specimens in the salt solution started turning slightly orange. Results are shown in Figure A2 of Appendix A.

The small dots may appear at the beginning of a degradation process with a nucleation as located in the bigger voids, from which the degradation extended. The brownish and beige tone could be a consequence of the integration of the salt and sugar adhesion to the surface. The transparency effect is also a symptom of degradation probably caused by a process of hydrolysis, where the dipolar water molecules tended to break the carbon hydrogen bonds of the polymer, as can be observed in Appendix A. As referred to the literature, the rearrangement of the polymeric chains in the printing procces [21] accelerates the hydrolytic degradation [22,23] as PLA and PETG as poysterers will be dissociated when they are introduced to water these groups. This disociation will happen first in distilled and salted water. Distilled water will disociate in OH^−^ and H^+^, and the salted solution in NaOH and HCl that easily reacts with the sters groups of the polymers. The sugar solution takes a longer period to disociate due to the stability of the solution.

## 4. Product Development Case Study

Once the behaviors of both materials in water environments have been analyzed, a case study of an oceanographic buoy is presented. PETG is used for the manufacture of this buoy, due to its low water absorption and low degradation, which allows it to maintain its dimensional stability and integrity.

**Figure 8:**
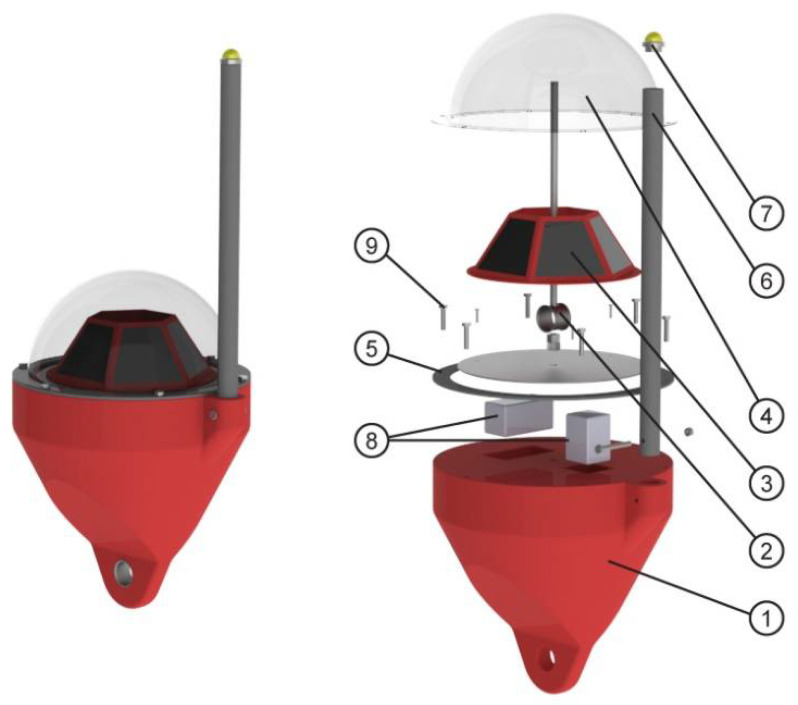
Exploded view of the oceanographic buoy, composed of (**1**) floating assembly, (**2**) galvanized steel insert system, (**3**) solar panel support, (**4**) transparent dome, (**5**) rubber seal, (**6**) mast, (**7**) beacon, (**8**) electronic equipment, and (**9**) set of screws and nuts.

The proposed product is an oceanographic buoy used to measure and transmit different environmental parameters such as wave periodicity, currents, temperature, and salinity (Figure 8). The product consists of the following components: a floatation assembly made with PETG by AM, a system of galvanized steel inserts that are responsible for transmitting the anchorage forces of mooring in a distributed way, a support for solar panels, also made of PETG by AM, a transparent dome also made of the same material and process, which allows the protection of the electronic equipment from the sea’s attacks and which in turn allows light to pass through, a rubber gasket, an HDPE (high density polyethylene) pipe mast, a beacon to indicate the position of the assembly, electronic equipment to collect and transmit data together with necessary batteries, and a set of screws and nuts also made of PETG and AM to prevent the corrosion of joints.

In the manufacture of this type of flotation elements, it is common to create a skin of polyethylene or other thermoplastics by rotomolding processes [47,48] or blow molding [49] and fill the interior with expanded polystyrene or polyurethane to achieve structural consistency under hydrostatic pressure [50]. The product developed in this work has been designed to be produced in a single process and with a single material, through the AM FFF.

**Figure 9:**
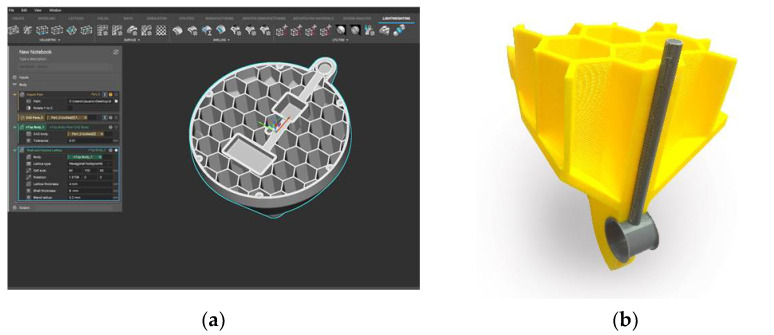
Sectioned views of the buoy: (**a**) Lattice design software for implicit design where the inner honeycomb structure has been done; (**b**) section of the mooring of the buoy made of PETG by fused filament fabrication (FFF).

The improvements introduced in relation to the original product are located in the main body of the buoy and in the dome. As depicted in Figure 9a, a section of the buoy is made of an outer skin and a honeycomb filling structure that provides rigidity and, in case of a collision and breakage of the outer skin, prevents water from entering the other compartments, thus ensuring buoyancy. Additionally, AM also allows the inclusion of metal inserts during the manufacturing process. A section of the buoy made of PETG using FFF is shown in Figure 9b. This image shows the metal insert inside and how it is embedded in the part. Likewise, the use of design tools for the AM of cellular structures allows the easy inclusion of a reinforcement assembly in the dome that improves its behavior on impact.

This product proposal shows an application of the absorption and degradation results obtained in this study. Concluding that the integration of this technology helps reduce manufacturing times and tooling costs and allows the production of a single piece, with a high degree of customization and adaptation to a specific industrial or scientific application.

## 5. Conclusions

In this paper, the evaluation of the degradation and absorption properties of additively manufactured specimens of PETG and PLA materials with FFF desktop printers is presented. Understanding their behavior and validating their use for product development in highly moisture environment such as maritime and food packing industries were obtained. The specimens have been immersed in two different fully saturated solutions of maritime salt and white sugar for 10 weeks to optically control the degradation and weight periodically in order to control water absorption until it was stable.

The results revealed that the main absorption of the tested specimens in the samples of both materials happened during the first three or four days. This absorption occurred in higher rates in specimens printed with FFF technologies than the ones produced with different industrial processes. This is explained by the multilayered construction approach of AM that creates internal microstructures in the printed parts with internal voids. This voids combined with the surface defects and the natural absorption rates of the material increased the amount of the absorbed solution. PETG specimens became stable in all three solutions, with subtle weight changes round 0.3% after 9 weeks, whereas PLA samples mean weight increase was 2.5% after 8 weeks. It is also remarkable that PLA presented an unstable character due to its organic nature that permits sudden changes in the absorption rates as the external surfaces degrade. Regarding the degradation, both materials experienced color variation caused by hydrolysis where dipolar water molecules tended to break the carbon hydrogen bonds of the polymer, and this color change was more exaggerated in the PLA specimens. In addition, the PETG specimens turned transparent probably due to a change in the crystallinity of the polymer. 

With these results, PETG has been selected as a suitable candidate for products and pieces that are in contact with liquid environments such maritime industry or packaging industry. Spare parts, molds, and tooling can be created. In this line, a case of study of the redesign, adapted to AM, of an existing buoy is presented by applying this material and improving its performance with weight reduction and improved water tightness. 

To completely understand the color and transparence changes, a deeper study about the modifications of the crystallinity of the polymers is required. Further research should be addressed for the quantification of the change in the mechanical properties, and real experimentation should be conducted with 3D printing products to validate the performance in these environments together with thermal analytical characterization for a better understanding of the materials nature.

## Figures and Tables

**Table 1:** Process parameters for printing specimens with polyethylene terephthalate glycol (PETG) and polylactic acid (PLA).

Process Parameters	PETG	PLA
Layer height (mm)	0.2	0.2
Printing rectilinear pattern (°)	0/90	0/90
Infill (%)	100	100
Printing temperature (°C)	260	210
Bed temperature (°C)	60	24
Printing speed (mm/s)	37.5	40
Adhesion type	Raft	Brim

## Data Availability

Not applicable.
